# Mental health complaints among healthcare workers engaged in the care of COVID‐19 patients: A prospective cohort study from Japan

**DOI:** 10.1002/jgf2.632

**Published:** 2023-06-20

**Authors:** Hiroki Namikawa, Yoshihiro Tochino, Akiko Okada, Keiko Ota, Yasuyo Okada, Koichi Yamada, Tetsuya Watanabe, Yasumitsu Mizobata, Hiroshi Kakeya, Yumiko Kuwatsuru, Toshihiko Shibata, Taichi Shuto

**Affiliations:** ^1^ Department of Medical Education and General Practice Osaka Metropolitan University, Graduate School of Medicine Osaka Japan; ^2^ Office of Institutional Research Osaka Metropolitan University, Faculty of Medicine Osaka Japan; ^3^ Center for Clinical Research and Innovation Osaka Metropolitan University Hospital Osaka Japan; ^4^ Department of Infection Control and Prevention Osaka Metropolitan University Hospital Osaka Japan; ^5^ Department of Infection Control Science Osaka Metropolitan University, Graduate School of Medicine Osaka Japan; ^6^ Department of Respiratory Medicine Osaka Metropolitan University, Graduate School of Medicine Osaka Japan; ^7^ Department of Traumatology and Critical Care Medicine Osaka Metropolitan University, Graduate School of Medicine Osaka Japan; ^8^ Department of Nursing Osaka Metropolitan University Hospital Osaka Japan; ^9^ Department of Cardiovascular Surgery Osaka Metropolitan University, Graduate School of Medicine Osaka Japan

**Keywords:** COVID‐19, healthcare workers, mental health, nurse, physical complaint, psychological complaint

## Abstract

**Background:**

Healthcare workers (HCWs) caring for patients with coronavirus disease‐2019 (COVID‐19) can experience physical and mental health burdens. It is imperative that hospitals reduce such burdens on frontline HCWs, protect them, and support their healthcare. This study aimed to investigate the association between occupation and the manifestation of physical or psychological symptoms among HCWs during the current COVID‐19 pandemic.

**Methods:**

A twice‐weekly survey using questionnaires targeting HCWs who care for COVID‐19 patients was performed at Osaka Metropolitan University Hospital (tertiary hospital). The demographic characteristics of the participants, exposure level, and physical and psychological complaints were evaluated.

**Results:**

Seventy‐one HCWs participated in this study, of whom 27 (38.0%) were doctors, 25 (35.2%) were nurses, and 19 (26.8%) were technicians. Among the HCWs, the proportions of those who experienced any physical or psychological symptoms were 28.2% and 31.0%, respectively. The frequency of depression and anxiety was obviously higher among the nurses than that among the doctors (both *p* < 0.01). Multivariate analysis revealed that being a nurse (odds ratio 4.90; *p* = 0.04) and having physical complaints (odds ratio 4.66; *p* = 0.02) might be independent predictors of the manifestation of psychological symptoms.

**Conclusion:**

Our results indicate that the follow‐up of HCWs experiencing physical symptoms, especially nurses engaged in the care of COVID‐19 patients, may require more careful management to improve the psychological outcomes. We believe that this study is the first step toward establishing a psychological health management strategy for HCWs caring for COVID‐19 patients.

## Graphical abstract

The prevalence of psychological symptoms in HCWs caring for patients with COVID‐19 was 31.0%. Being a nurse and having physical complaints might be independent predictors of the manifestation of psychological symptoms from caring for COVID‐19 patients. The follow‐up of HCWs experiencing physical symptoms, especially nurses engaged in the care of COVID‐19 patients, may require more careful management to improve the psychological outcomes.

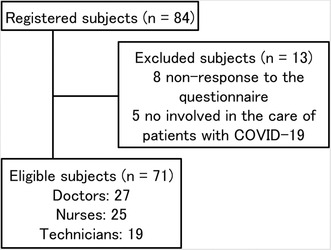

## 1 INTRODUCTION

The novel coronavirus disease‐2019 (COVID‐19) first emerged in Wuhan, Hubei Province, China, in December 2019.^1^ It rapidly spread to other countries, causing a pandemic.^2^ Healthcare workers (HCWs) are in high demand, as they play a critical role in responding to the pandemic.^3^ However, they can experience physical and mental health burdens due to a continued increase in the number of infected and suspected cases, heavy workload, lack of personal protective equipment, excessive media coverage, and concerns about inadequate support.^4^ Particularly, the psychological impact of COVID‐19 on HCWs is remarkable, with high levels of depression, anxiety, insomnia, and distress being reported.^5^ During this crisis, the physical and mental health problems frontline HCWs face should be regarded as a pressing global public health concern. In addition, it is imperative that hospitals reduce both the physical and mental burdens of frontline HCWs, protect them, and support their healthcare.

Previously, we conducted a study to investigate the association between occupation and the manifestation of physical symptoms among HCWs caring for critically ill patients with COVID‐19 pneumonia at a tertiary hospital in Japan and did not include psychological problems in the survey items.^6^ Several studies have reported the management of physical or mental health among HCWs during the COVID‐19 pandemic.^7^, ^8^, ^9^ Furthermore, Shaukat et al.^10^ showed that frontline HCWs providing care to patients with COVID‐19 experienced varying degrees of physical and psychological impacts depending on their occupation.

However, to the best of our knowledge, no prospective studies have focused on both the physical and mental health of medical workers caring for patients with COVID‐19. The aim of this study was to investigate the association between occupation and the manifestation of physical or psychological symptoms among HCWs in Japan during the current COVID‐19 pandemic.

## 2 METHODS

### 2.1 Study setting, population, and procedure

**FIGURE 1:**
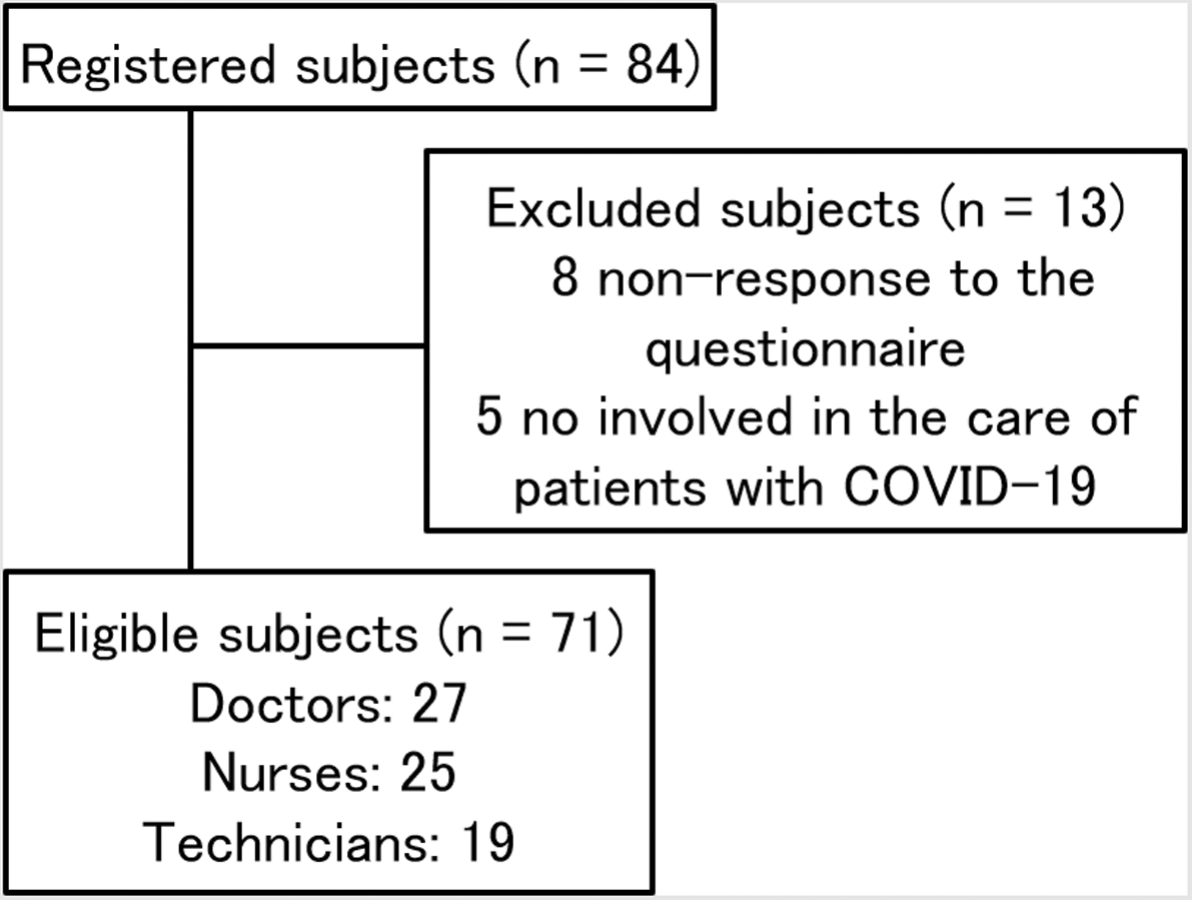
The present study flowchart diagram. Eighty‐four HCWs were enrolled using the REDCap tool. Seventy‐one HCWs were eligible for this study, and 13 HCWs were excluded from this study. Of the 71 HCWs, 27 were doctors, 25 were nurses, and 19 were technicians.

This prospective cohort study was performed from December 1, 2020 to February 28, 2021, using a shareable Research Electronic Data Capture (REDCap) tool.^11^ During this study period, HCWs were actively involved in the care of COVID‐19 patients who were admitted to Osaka Metropolitan University Hospital (tertiary hospital) or a municipal hospital specializing in COVID‐19 management. Patients with severe COVID‐19 (admitted to the intensive care unit and required mechanical ventilation)^12^ were admitted to Osaka Metropolitan University Hospital, while patients with mild or moderate COVID‐19 (not severe) were admitted to a municipal hospital specializing in COVID‐19 management. The study participants included doctors, nurses, and technicians (radiological, biomedical equipment, and clinical microbiology laboratory technicians). Some of the doctors were involved in the care of COVID‐19 patients who were admitted to a municipal hospital. This research used convenience sampling strategies. We used the same method as that in our previous study to deliver the questionnaire to the participants.^6^ Questionnaires were delivered to the study participants twice a week via REDCap. When the study participants did not provide patient care for 14 consecutive days, we stopped delivering the questionnaire to them. Finally, we targeted those who completed these questionnaires. The present study flowchart diagram is shown in Figure 1. This study was approved by the Ethics Committee of Osaka Metropolitan University (approval number 2020‐170). The need for written informed consent was waived owing to the clinical research using opt‐out.

### 2.2 Screening questionnaire items

As described in our previous study, the questionnaire was prepared with a slight modification.^6^ The questionnaire briefly assessed three main components: demographic characteristics, exposure level, and postexposure physical/psychological complaints. Demographic characteristics included age, gender, occupation (doctor, nurse, or technician), and workplace. Exposure was assessed according to the degree (high‐ or low‐risk contact) and frequency (days a week, 1–7). We defined high‐risk contact as entering a hospital room and having direct contact with COVID‐19 patients, and low‐risk contact as not entering a hospital room but being involved with COVID‐19 patients in some way. Physical complaints comprised attention‐requiring and observation‐requiring symptoms (yes/no answers). Attention‐requiring symptoms included fever, intense fatigue, dyspnoea, and dysosmia or dysgeusia. Observation‐requiring symptoms included sore throat, myalgia or arthralgia, headache, cough/sputum production, diarrhea or stomach ache, nasal discharge or sneeze or nasal obstruction, congestion or ophthalmalgia or low vision, and rash. Psychological complaints included depression, anxiety, insomnia, distress, and nervousness. We also confirmed whether the participants required an internal medical examination.

### 2.3 Study outcomes

We evaluated the prevalence of physical and psychological complaints postexposure reported by the HCWs. Additionally, we investigated the association between occupation and the manifestation of psychological symptoms among the HCWs. Experiencing at least one of the above psychological complaints at least once during this research period was defined as the manifestation of psychological symptoms. We prospectively collected outcome variables for each response to the twice‐weekly survey during this study.

### 2.4 Statistical analyses

Participant characteristics, exposure, and physical/psychological complaints prospectively collected were compared between the doctor, nurse, and technician groups. Fisher's exact test and the Kruskal–Wallis test were used for the univariate comparison of categorical data. Additionally, the Bonferroni correction method was used for *p* value adjustment. To determine the independent predictors of the manifestation of psychological symptoms, variables determined to be clinically important based on prior studies were considered for inclusion in the multivariate logistic regressions.^5^ All statistical analyses were performed with EZR on the R software.^13^ EZR is a modified version of the R commander, which includes the statistical functions frequently used in biostatistics. A *p* value of <0.05 was considered statistically significant.

## 3 RESULTS

During our study period, the total number of full‐time HCWs (doctors, nurses, and technicians) affiliated with Osaka Metropolitan University Hospital was 1490. Finally, 84 HCWs were enrolled in the REDCap, 71 of whom were eligible for this study. Thirteen HCWs were excluded from this study: eight HCWs did not respond to the questionnaire and five were not involved in the care of patients with COVID‐19. The baseline characteristics of the participants who were eligible for this study are summarized in Table 1. Of the 71 HCWs, 27 (38.0%) were doctors, 25 (35.2%) were nurses, and 19 (26.8%) were technicians (radiological: 15, biomedical equipment: 2, clinical laboratory: 2). The participants reported a mean of 4.2 maximum days of exposure per week. Twenty HCWs (28.2%) had physical complaints; the main symptoms included headache (11.3%) and nasal symptoms (18.3%). Twenty‐two HCWs (31.0%) had psychological complaints; the main symptoms included anxiety (25.4%) and nervousness (23.9%). A comparison of the baseline characteristics between occupations is summarized in Table 2. There were significant differences in age (*p* = 0.03), gender (*p* < 0.001), workplace (*p* < 0.001), frequency of exposure (*p* = 0.001), psychological complaints (*p* = 0.02), depression (*p* < 0.001), anxiety (*p* < 0.001), and nervousness (*p* = 0.02) among the three groups. Additionally, the nurses were noticeably younger than the doctors (nurse: 32.3 ± 8.6 years, doctor: 38.0 ± 7.0 years, *p* = 0.03), the nurses had a higher frequency of exposure than that among the doctors and technicians (nurse: 5.0 ± 1.1, doctor: 3.7 ± 1.5, technician: 3.8 ± 1.0, nurse vs doctor or technician, both *p* < 0.01), and the frequency of depression and anxiety was obviously higher among the nurses than that among the doctors (depression: nurse 36.0%, doctor 0%, anxiety: nurse 52.0%, doctor 7.4%, both *p* < 0.01). Meanwhile, the frequency of physical complaints did not differ between the three groups. Multivariate analysis (explanatory variables: age, male, nurse, exposure frequency, and physical complaint) revealed that being a nurse (odds ratio 4.90; *p* = 0.04) and having a physical complaint (odds ratio 4.66; *p* = 0.02) might be independent predictors of the manifestation of psychological symptoms (Table 3).

**TABLE 1:** Baseline characteristics of the study participants.

Variables	
Age (years)^a^	35.4 ± 8.0
Gender (male/female)	38/33
Occupation	
Doctor	27 (38.0%)
Nurse	25 (35.2%)
Technician	19 (26.8%)
Workplace	
Tertiary	51 (71.8%)
Municipal	20 (28.2%)
Exposure	
Degree	
High risk	67 (94.4%)
Low risk	4 (5.6%)
Frequency (days a week)	
1	3 (4.2%)
2	6 (8.4%)
3	12 (16.9%)
4	18 (25.4%)
5	21 (29.6%)
6	9 (12.7%)
7	2 (2.8%)
Physical complaint	20 (28.2%)
Attention‐requiring symptoms	1 (1.4%)
Fever	0 (0%)
Intense fatigue	1 (1.4%)
Dyspnoea	0 (0%)
Dysosmia or dysgeusia	0 (0%)
Observation‐requiring symptoms	20 (28.2%)
Sore throat	3 (4.2%)
Myalgia or arthralgia	2 (2.8%)
Headache	8 (11.3%)
Cough or sputum production	2 (2.8%)
Diarrhea or stomach ache	5 (5.6%)
Nasal discharge or sneezing or nasal obstruction	13 (18.3%)
Congestion or ophthalmalgia or low vision	7 (9.9%)
Rash	2 (2.8%)
Psychological complaint	22 (31.0%)
Depression	11 (15.5%)
Anxiety	18 (25.4%)
Insomnia	10 (14.1%)
Distress	5 (7.0%)
Nervous	17 (23.9%)
Applicant for internal medical examination	0 (0%)

^a^ Data are presented as means ± standard deviation.

**TABLE 2:** Comparison of baseline characteristics between occupations.

Variables	Doctor (*n* = 27)	Nurse (*n* = 25)	Technician (*n* = 19)	*p* Value^a^
Age (years)^b^	38.0 ± 7.0	32.3 ± 8.6	35.9 ± 6.8	0.03^c^
Male	21 (77.8%)	4 (16.0%)	13 (68.4%)	<0.001^d^
Workplace				
Tertiary	7 (25.9%)	25 (100.0%)	19 (100.0%)	<0.001^e^
Exposure				
Degree				
High risk	25 (92.6%)	25 (100.0%)	17 (89.5%)	0.30
Frequency (days a week)^b^	3.7 ± 1.5	5.0 ± 1.1	3.8 ± 1.0	0.001^f^
Physical complaint	8 (29.6%)	8 (32.0%)	4 (21.1%)	0.75
Attention‐requiring symptoms	0 (0%)	1 (4.0%)	0 (0%)	0.62
Fever	0 (0%)	0 (0%)	0 (0%)	NA
Intense fatigue	0 (0%)	1 (4.0%)	0 (0%)	0.62
Dyspnoea	0 (0%)	0 (0%)	0 (0%)	NA
Dysosmia/Dysgeusia	0 (0%)	0 (0%)	0 (0%)	NA
Observation‐requiring symptoms	8 (29.6%)	8 (32.0%)	4 (21.1%)	0.75
Sore throat	2 (7.4%)	1 (4.0%)	0 (0%)	0.78
Myalgia/arthralgia	0 (0%)	1 (4.0%)	1 (5.3%)	0.52
Headache	1 (3.7%)	5 (20.0%)	2 (10.5%)	0.20
Cough/sputum production	1 (3.7%)	0 (0%)	1 (5.3%)	0.73
Diarrhea/stomach ache	0 (0%)	3 (12.0%)	1 (5.3%)	0.13
Nasal discharge/sneeze/nasal obstruction	6 (22.2%)	4 (16.0%)	3 (15.8%)	0.86
Congestion/ophthalmalgia/low vision	3 (11.1%)	3 (12.0%)	1 (5.3%)	0.78
Rash	0 (0%)	2 (8.0%)	0 (0%)	0.19
Psychological complaint	5 (18.5%)	13 (52.0%)	4 (21.1%)	0.02^g^
Depression	0 (0%)	9 (36.0%)	2 (10.5%)	<0.001^h^
Anxiety	2 (7.4%)	13 (52.0%)	3 (15.8%)	<0.001^i^
Insomnia	1 (3.7%)	5 (20.0%)	4 (21.1%)	0.14
Distress	1 (3.7%)	4 (16.0%)	0 (0%)	0.15
Nervousness	4 (14.8%)	11 (44.0%)	2 (10.5%)	0.02^j^
Applicant for internal medical examination	0 (0%)	0 (0%)	0 (0%)	NA

Abbreviation: NA, not available.

^a^ Continuous variable: Kruskal–Wallis test with the Bonferroni correction method. Categorical variable: Fisher's exact test with the Bonferroni correction method.

^b^ Data are presented as means ± standard deviation.

^c^ Doctor versus Nurse, *p* = 0.03, Doctor versus Technician, *p* = 1.0, Nurse versus Technician, *p* = 0.41.

^d^ Doctor versus Nurse, *p* < 0.001, Doctor versus Technician, *p* < 0.01, Nurse versus Technician, *p* < 0.001.

^e^ Doctor versus Nurse, *p* < 0.001, Doctor versus Technician, *p* < 0.001, Nurse versus Technician, *p* = 1.0.

^f^ Doctor versus Nurse, *p* < 0.01, Doctor versus Technician, *p* = 1.0, Nurse versus Technician, *p* < 0.01.

^g^ Doctor versus Nurse, *p* = 0.06, Doctor versus Technician, *p* = 1.0, Nurse versus Technician, *p* = 0.18.

^h^ Doctor versus Nurse, *p* < 0.01, Doctor versus Technician, *p* = 0.5, Nurse versus Technician, *p* = 0.24.

^i^ Doctor versus Nurse, *p* < 0.01, Doctor versus Technician, *p* = 1.0, Nurse versus Technician, *p* = 0.08.

^j^ Doctor versus Nurse, *p* = 0.10, Doctor versus Technician, *p* = 1.0, Nurse versus Technician, *p* = 0.06.

**TABLE 3:** Multivariate analysis of predictors associated with the manifestation of psychological symptoms.

Predictors	OR (95% CI)	*p* Value
Age	1.05 (0.97–1.13)	0.24
Male	0.82 (0.20–3.41)	0.79
Nurse	4.90 (1.04–23.1)	0.04
Exposure frequency	1.11 (0.71–1.74)	0.64
Psychological complaint	4.66 (1.34–16.2)	0.02

Abbreviations: CI, confidence interval; OR, odds ratio.

## 4 DISCUSSION

Our prospective cohort study of 71 HCWs caring for patients with COVID‐19 revealed that 31.0% of such HCWs developed psychological symptoms. It was also suggested that being a nurse and having physical complaints may be independent predictors of the manifestation of psychological symptoms from caring for COVID‐19 patients. Several studies have reported that the manifestation of psychological symptoms among frontline HCWs caring for COVID‐19 patients was associated with being a nurse or physical complaints.^9^, ^10^, ^14^, ^15^ Compared to the world's HCW population living in the ongoing pandemic in 2023, the number of respondents in this study was so small that the results may not be considered representative. However, based on the results of the previous studies mentioned above, we believe that there is a close relationship between these factors and discuss them in more detail.

Healthcare workers work long hours under high pressure during the pandemic, and it is well known that they are subjected to a strong psychological impact due to COVID‐19. Previous studies have reported the frequency of psychological symptoms among HCWs during the pandemic.^4^, ^9^, ^16^, ^17^, ^18^ The most common studied outcomes were depression and anxiety. The prevalence of depression and anxiety ranged between 8.9%–50.4% and 8.3%–44.6%, respectively. Previous studies showed that medical HCWs (doctors and nurses) had significantly higher levels of psychological distress, including depression and anxiety, compared with those among nonmedical HCWs (administrative staff).^17^, ^19^ Furthermore, previous studies reported that frontline workers or HCWs in direct contact with COVID‐19 patients also had higher levels of psychological distress including depression and anxiety.^4^, ^17^, ^20^ In our study as well, the frequency of psychological complaints among HCWs was relatively high at 31.0%, with depression and anxiety at 15.5% and 25.4%, respectively. These findings indicate that occupation is an important risk factor associated with adverse mental health outcomes among HCWs during the COVID‐19 pandemic.

Notably, previous studies showed that nurses might be at a significant risk of more mental health problems than that of doctors during the COVID‐19 pandemic.^4^, ^21^, ^22^ There are several possible reasons for these findings. First, frontline nurses caring for COVID‐19 patients have much more direct and intensive contact with patients than that by other HCWs^4^ which increases their risk of infection^23^, ^24^ and leads to poor mental health.^20^ Our study also showed that the nurses had a higher frequency of exposure than that of the doctors and technicians. Second, nurses had more junior titles (fewer years of work experience) than other HCWs, and this inexperience can lead to psychological distress.^4^ Our study also showed that the nurses were younger than other HCWs, and it was possible that a relatively large number of nurses with junior titles were engaged in the care of COVID‐19 patients. Third, Cai et al.^21^ reported that nurses had more concerns about extra financial compensation during or after the COVID‐19 outbreak and felt significantly more nervous and anxious at the wards compared with those in other HCWs. These findings suggest that being a nurse may be associated with an increased risk of the manifestation of psychological symptoms from caring for COVID‐19 patients. However, the aforementioned result needs to be interpreted cautiously, as the measurements for the intensity of exposure are very crude. The patients need to be assessed more comprehensively to explore what aspects of a nurse's experience make them at risk in a future study.

Previous studies reported that the HCWs involved in the care of COVID‐19 patients experienced various physical symptoms^6^, ^9^ and can develop psychological sequelae.^25^ Chew et al.^9^ reported that the psychological impact of the COVID‐19 outbreak may contribute to the increase in self‐reported physical symptoms. On the other hand, Yang pointed to the possibility that the social stigma associated with mental illness may increase the tendency to report psychological distress as physical symptoms.^26^ It may be difficult to determine the true relationship between physical symptoms and psychological symptoms. However, Chew et al.^9^ reported the bi‐directional complex association between psychological outcomes and physical symptoms, where nonattention‐requiring psychological distress led to the exacerbation of physical symptoms among HCWs during the COVID‐19 outbreak. Thus, these findings suggest that physical complaints may be an independent predictor of the manifestation of psychological symptoms from caring for COVID‐19 patients. Therefore, it is very important that we support the HCWs engaged in the care of COVID‐19 patients, addressing both psychological and physical health burdens.

The present study has several limitations. First, the study population was relatively small, and the study involved HCWs caring for patients with COVID‐19 only in two hospitals; therefore, there was selection bias. Future studies including more HCWs caring for COVID‐19 patients in multinational, multicenter settings are required to address this limitation. Additionally, physical and psychological assessments were based on a self‐reported online survey using the REDCap tool. A more detailed assessment of the physical and psychological problems is required in future studies. Third, this study was conducted over a short duration of 3 months, so no long‐term follow‐up was done afterward. The various symptoms experienced by HCWs caring for patients with COVID‐19, especially psychological symptoms, can accumulate over time and become more severe. Therefore, there is a need to conduct long‐term research on these symptoms. Fourth, we have only assessed two aspects of exposure: degree (high‐ or low‐risk contact) and frequency (days a week). In the future, it will be necessary to investigate the procedures regarding the care of COVID‐19 patients and contact duration. Fifth, the data collection for this study was performed more than 2 years ago. As the perception of psycho‐physical stress toward novel coronavirus has evolved dramatically over this period, some of the findings may have become outdated. Finally, this study did not control the effect of psychological symptoms at baseline. The psychological symptoms at baseline may be the most powerful predictor of psychological symptoms during the follow‐up period. We will need to check the psychological symptoms at baseline in future studies.

## 5 CONCLUSIONS

In summary, the present study demonstrated that the prevalence of psychological symptoms in HCWs caring for patients with COVID‐19 was 31.0%. Being a nurse and having physical complaints may be independent predictors of the manifestation of psychological symptoms from caring for COVID‐19 patients. Therefore, the follow‐up of the HCWs experiencing the physical symptoms, especially nurses, may require more careful management to improve the psychological outcomes. We believe that this study is the first step toward establishing a psychological health management strategy for HCWs caring for COVID‐19 patients.

## AUTHOR CONTRIBUTIONS

HN, YT, and T. Shibata designed this study. AO and KO set up the survey system. YO, KY, TW, YM, HK, YK, and T. Shibata conducted infection management in our hospital. HN, YT, and T. Shuto conducted the clinical interpretation. HN and YT drafted the manuscript and critically revised it. All authors contributed to the final version of the manuscript and approved its submission.

## FUNDING INFORMATION

None.

## CONFLICT OF INTEREST STATEMENT

All authors declare no conflict of interest.

## ETHICS APPROVAL STATEMENT

This study was approved by the Ethics Committee of Osaka Metropolitan University (approval number 2020‐170).

## PATIENT CONSENT STATEMENT

The purpose of this questionnaire was to manage the health of HCWs, which was different from the research purposes. Therefore, the need for written informed consent was waived due to the clinical research using opt‐out. This has been approved by the Ethics Committee of Osaka Metropolitan University.

## CLINICAL TRIAL REGISTRATION

None.

## CONSENT FOR PUBLICATION

All authors have approved the manuscript for submission.

## Data Availability

The data that support the findings of this study are available from the corresponding author upon request.
